# Polish High School Students’ Knowledge about Cancer

**DOI:** 10.3390/ijerph18094765

**Published:** 2021-04-29

**Authors:** Monika Rucinska, Radoslaw Sroda, Olga Wilk, Arian Saied, Jakub Miloszewski, Anna Sugajska, Karolina Osowiecka

**Affiliations:** 1 Department of Oncology, Collegium Medicum, University of Warmia and Mazury in Olsztyn, 10-228 Olsztyn, Poland; m_rucinska@poczta.onet.pl (M.R.); anna.sugajska@gmail.com (A.S.); 2 Student Science Club, Department of Oncology, Collegium Medicum, University of Warmia and Mazury in Olsztyn, 10-228 Olsztyn, Poland; sr.radoslaw@gmail.com (R.S.); olgawilk2@gmail.com (O.W.); arisai@wp.pl (A.S.); kubamiloszewski@onet.pl (J.M.); 3 Department of Psychology and Sociology of Health and Public Health, School of Public Health, University of Warmia and Mazury in Olsztyn, 11-041 Olsztyn, Poland

**Keywords:** cancer risk factor, high school students, knowledge

## Abstract

Background: Cancer, as the second most common cause of death after cardiovascular diseases, is a global health problem. There is still an increasing number of cancer incidences and deaths. Methods: The study was conducted as a part of the health promotion educational project concerning oncological education to develop the knowledge of cancer risk factors among high school students in Poland. A special questionnaire was filled out by students before the educational lesson on cancer conducted by medical students and young doctors. Results: The study was carried out on 227 high school students (aged 17–18 years). Most students (67.5%) indicated that genetic predisposition is the most important cancer risk factor. Only about a quarter of students pointed to the relationship between lifestyle and cancer. Moreover, 41% of students admitted to smoking cigarettes. Most of them (80.6%) claimed that they can modify their own cancer risk. Almost all responders believed that early detected cancer is curable. Conclusions: High school students do not know about cancer risk factors and they do not relate cancer with lifestyle. Some students indicated bad lifestyle habits such as tobacco smoking. It is necessary to emphasize cancer prevention in early education, especially focusing on modification of lifestyle.

## 1. Introduction

Cancer, as the second most common cause of death after cardiovascular diseases, is a global health problem. There is still an increasing number of cancer incidences and deaths in the world, and also in Poland. In 2018, more than 167 thousand new cases and 100 thousand deaths were noted [1]. The World Health Organization estimates that the number of new cases worldwide will reach 21.6 million in 2030, and cancer deaths will be above 12 million in 2030 [2]. Public knowledge about cancer is important to limit the increase in incidences. Awareness of cancer risk factors should be developed from a young age to better control their health through a healthy lifestyle. The concept of the “health field” in Lalonde’s report [3] is considered to be composed of four interdependent fields determined to influence an individual’s health, including lifestyle (50%), environmental (20%), biology (20%), and health care organization (10%) factors. About half of incidences of cancer are dependent only on behavioral habits like smoking cigarettes, improper diet, drinking alcohol, low physical activity, and risky sexual behavior [4]. Behavior change may significantly affect the rate of cancer incidents. Changes in lifestyle at a young age are easier and have an influence on health in the future, so awareness about cancer should be expanded as soon as it is possible. A healthy lifestyle and avoiding cancer risk factors are possible if people have the knowledge and understanding of how their behavior will affect their health in the future. However, little is known about adolescents’ knowledge of cancer and its risk factors.

The aim of this study was to determine the knowledge about cancer factor risks among Polish high school students.

## 2. Materials and Methods

The study was conducted as a part of the health promotion educational project provided by medical students and young doctors from the Students Science Club and the Department of Oncology, University of Warmia and Mazury in Olsztyn, Poland. This project concentrated on oncological education to develop the knowledge of cancer risk factors among high school students. Special lessons were prepared on the epidemiological and etiological problems related to cancer. The content of the lessons was designed by medical students in cooperation with an oncologist. Lessons were conducted by medical students and young doctors. The participation in this project was proposed for all 10 high schools in Olsztyn, Poland. Five of them agreed to cooperate. At the time of study (2017–2018), high school education in Poland consisted of 3 years. The research was done among second year students.

A questionnaire prepared especially for this study was used (Supplementary Materials). The questionnaire was designed specifically for this study in accordance with general principles. The questionnaire consisted of 8 main closed-ended quantitative questions and 3 questions concerning demographic data (age, gender, place of residence). The reliability of the questionnaire was not assessed. The comprehensibility and acceptability of the questionnaire was validated by the psycho-oncologist. After the first twenty students filled out the questionnaire, they were interviewed to verify they properly understood the questions. The study was carried out between April 2017 and April 2018. Students filled out the questionnaire before the educational lesson. Participation in the study was voluntary and anonymous.

### Statistical Analysis

Descriptive statistics (percentages, median, and interquartile range) were estimated. The proportion in the analyzed subgroups was compared using the chi-square test. The Mann–Whitney test was conducted for a comparison of medians.

To estimate the subjective student’s knowledge about cancer before the educational lesson the 4-point Likert scale was used (1—bad, 2—moderate, 3—good, 4—very good). A *p*-value < 0.05 was considered to be statistically significant. The analysis was conducted using TIBCO Software Inc., Krakow, Poland (2017). Statistica (data analysis software system), version 13. http://statistica.io (accessed on 1 April 2020).

## 3. Results

The study was conducted on a group of 227 students (aged 17–18 years) from 5 high schools in Olsztyn. There were 125 females and 101 males (one unknown). Most of them lived in a city (80.6%) (Table 1).

Almost 80% of students knew the definition of cancer and this was not associated with sex or place of residence. However, students living in cities more frequently indicated the right definition of cancer (*p* = 0.08; Χ^2^ test) (Table 2).

**Figure 1:**
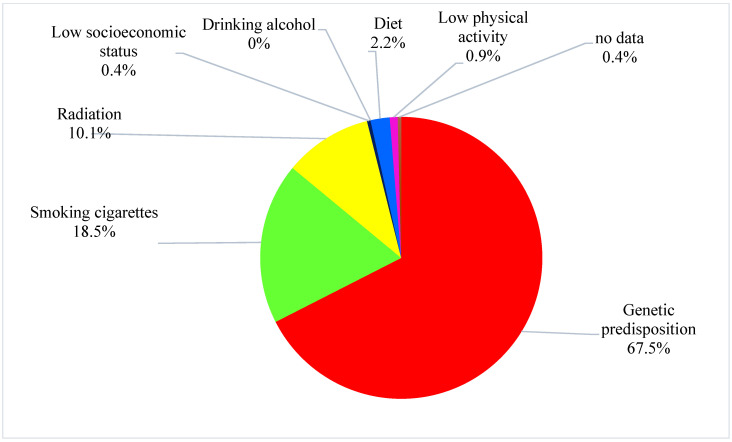
The students’ opinion about the most important cancer risk factor.

Most students (67.5%) indicated that genetic predispositions are the most important cancer risk factor. Only about a quarter of students (22%) pointed to the relationship between lifestyle and cancer. Less than a fifth of students (18.5%) indicated that smoking cigarettes is a cancer risk factor. No one thought that drinking alcohol could be a cause of cancer and only a handful of people related diet (2.2%) or low physical activity (0.9%) to cancer (Table 2, Figure 1).

Statistically, females more often claimed that the main factor causing cancer is genetic predisposition. Males almost twice as many times as females indicated lifestyle and radiation as main cancer risk factors (*p* = 0.02; Χ^2^ test). There was no significant difference associated with place of residence (Table 2).

Although students indicated genetics as the most significant in cancer development, most of them (80.6%) claimed that they can modify their own cancer risk independently of what they indicated as a major cancer risk factor (*p* = 0.36; Χ^2^ test). Sex and place of residence also had no impact on this (Table 2).

More than half of responders (58%) claimed that they lived a healthy lifestyle. However, 41% of students admitted to smoking cigarettes (including students who used electronic cigarettes). Moreover, 40% of students’ parents were noted as smokers, but there was no significant correlation between the smoking behaviors of students and their parents (*p* = 0.06; Χ^2^ test) (Table 2). The relation between smoking status and a healthy lifestyle was noted. Most of the students (69%) who admitted to not smoking cigarettes indicated also having healthy behaviors, and more than half of smokers (58%) did not confirm leading a healthy lifestyle (*p* < 0.001; Χ^2^ test).

Almost all responders, except eight, believed that early detected cancer is curable (96.5%). Factors such as sex and place of residence did not influence students’ perception (*p* > 0.05; Χ^2^ test) (Table 2).

Before the educational lesson focused on problems related to cancer, students estimated their knowledge about cancer at a moderate level—at a median of 2 (95% IQR 2–3) on a 4-point scale. There was no significant correlation between subjective assessment of students’ knowledge about cancer and sex or place of residence (*p* > 0.05; Mann–Whitney test).

## 4. Discussion

Developing proper health habits in adolescence should lead to the maintenance of good health in adulthood. Some authors underlined that high school and undergraduate students have poor knowledge about cancer risk factors [5,6,7,8,9,10,11,12,13,14]. The awareness of the risk factors gives the chance to avoid them and thus reduces the risk of cancer. In Poland, there is a lack of studies on the attitudes and knowledge of cancer risk factors among high school students. There is only one paper, which determined that Polish high school students did not have adequate knowledge of head and neck cancer, as well as its risk factors and prevention [5]. We showed that young people in Poland, especially those living in cities, defined cancer properly. However, in our study, students were not very knowledgeable about cancer risk factors, for example, only less than a fifth of students indicated that smoking cigarettes is a cancer risk factor. No one thought that drinking alcohol could be a cause of cancer. Our questions were about cancer in general. According to the study conducted in Poland by Wnuk et al. [5], more students indicated that tobacco smoking was a head and neck cancer risk factor than in our research (87.3% vs. 18.5%). In contrast to our study, about a quarter of students from the Wnuk et al. analysis [5] considered that excessive alcohol consumption can also cause cancer. This difference could come from the fact that in our study Polish students were asked about cancer in general, while in the Wnuk et al. study [5], they were asked about oral cancer. It is clearer that this kind of cancer is caused by cigarettes/alcohol consumption. High school students more often declared these risks in comparison with technical school students [5], maybe because they have a more general education. Moreover, adolescents frequently identified smoking as a risk factor for lung cancer in comparison to head and neck cancer (82.4–99% vs. 15.7–65%) [15,16]. Makwe et al. [11] showed that 22.5% of students declared that smoking increases the risk of cervical cancer. Although both adults’ and adolescents’ knowledge about cancer risk factors was poor, they indicated smoking at the top of cancer risk factors’ list, and they especially associated smoking with lung cancer [17,18,19,20]. A cross-sectional survey of 2100 adult residents in England showed that more than 50% of individuals are unaware of the alcohol–cancer link [21], which is consistent with our results on Polish students, who did not recognize alcohol as a risk factor for cancer at all.

Imran et al. [22] showed that 66% of students knew that colorectal cancer is related to obesity and lack of exercise. In the case of head and neck cancer, only 22% of high school students pointed to the relationship between lifestyle and cancer [5]. Fewer than 50% of students thought that breast cancer could be related to lifestyle, for example, obesity [6]. Only 15% of students claimed that eating red meat and 7% that low consumption of fruits and vegetables increased cancer risk, and low levels of physical activity were identified by a quarter of adolescents [18]. In our study, the percentage was even lower—only a handful of people related diet (2%) or low physical activity (0.9%) with cancer. Merten et al. [16] showed that adults’ awareness of the relationship between a high fat diet and breast cancer and endometrial cancer was also low (44% and 25%, respectively). Only about 10–15% of British adults linked a high fat diet and low fruits and vegetables diet with breast cancer and 33–42% with colorectal cancer [17].

In the case of breast cancer, the most known risk factor among high school and university students was a family history of breast cancer. Students were not aware of the long-term risk posed by oral contraceptives, hormone replacement therapy, or the potential benefits of breast feeding and early childbirth [13,14]. In the study about knowledge and awareness of colorectal cancer, 52% of students pointed to family history as a risk factor [22]. Moreover, adults think that family history of cancer is an important factor. In the study that included 748 Irish adults, 26% believed that over 50% of cancers are inherited and almost half of respondents listed genetics as one of the main risks [19]. In the study by Karadeniz et al. [20], history of cancer as a risk factor was indicated by 83% of individuals. A high percentage (67.5%) of the Polish students in our study also claimed that the genetic factor was the most important risk factor for cancer. Statistically, females more often pointed to genetic factors, whereas males indicated factors related to lifestyle as a cancer risk. This difference is difficult to explain.

It is difficult to talk about early prevention if people generally do not know the risk factors. It seems necessary to be aware that the cancer risk can be reduced by the individuals themselves. The Ryan et al. study [19] showed that about 10% of adults believed it is impossible to modify cancer risk. High school students in our study also thought that people have an influence on cancer development. However, we observed some inconsistencies in youth reasoning about cancer. Most of the Polish students believed that the genetic factor has the most important influence on cancer development, but on the other hand, 80.6% of students claimed that people can modify their own cancer risk. It is probable that the source of their knowledge was not from reliable sources, but from the media/internet and students did not understand this information properly. Karayurt et al. [13] showed that students turned out to know little about lifestyle changes, such as diet, smoking, and alcohol use. It is likely that young people do not understand exactly what a healthy lifestyle is. In our study, 41% of Polish high school students admitted to smoking cigarettes but claimed that they have lived a healthy lifestyle. The percentage of smoking among adolescents in our study was very high. Moreover, Wnuk et al. [5] showed that an enormous number of Polish students had bad lifestyle habits—tobacco smoking (29.1% of students smoke or vape), alcohol consumption, as well as poor oral hygiene. Moreover, technical school students smoke more often (43.3%) and consume more alcohol (67%) than standard high school students (22.9% and 52.5%, respectively). Nowak et al. [23] showed that 37.6% of 535 secondary school students in Poland aged 13–17 years smoke cigarettes and 36.1% of them consume alcohol. Europe has a high prevalence of cigarette smoking among adolescents, but this differs across countries (from 5% in Armenia to 51% in Greenland) [24]. The prevalence of smoking is also high among school students in Saudi Arabia, including 20% current smokers and 16% ex-smokers [25]. By contrast, in the US, smoking is declared by only 10% of adolescents [26]. Frequent cigarette smoking among adolescents is worrying because smoking was noted as the strongest lifestyle related risk factor overall and may be responsible for about 20–30% of all incidents of cancer. It is assumed that half of all cancers could be avoided by not smoking, reducing alcohol drinking, proper dieting, and engaging in physical activity [27]. Students should be aware that lifestyle changes—such as quickly giving up smoking and drinking alcohol—can help to prevent cancer in the future.

In our study, almost all responders (96.5%) believed that in general early detected cancer is curable. The majority of undergraduate students in Saudi Arabia thought that colorectal cancer is a disease that can be cured if detected early [22], but only 50% of high school students agreed that early detection of breast cancer enhances the chances of recovery [6].

Before the educational lesson focused on problems related to cancer, Polish students estimated their knowledge about cancer at a moderate level—at a median of 2 (95% IQR 2–3) on a 4-point scale. It seems to be necessary to teach young people about cancer risk factors. This could help students to better understand cancer and encourage them to modify their lifestyle [28]. Kyle et al. [18] presented results of a cancer-specific school-based educational intervention for adolescents provided by Teenage Cancer Trust, causing a significant increase in the awareness of lifestyle factors related to cancer risk. Adequate education is important to reduce false beliefs. In Ryan’s study [19], 12% of adult participants believed that luck is important in avoiding cancer. The participants thought that if someone in their family developed cancer, they could do nothing to reduce their personal cancer risk [19].

### Limitation of the Study

The study has some limitations. The size of the sample was relatively small. To get more valuable data, the questionnaire should include some extra checking questions. Since the sample of the study included only students from high schools in one city, the results cannot be generalized. 

## 5. Conclusions

High school students in Poland do not know cancer risk factors and do not relate cancer development with lifestyle. Additionally, a number of students indicated bad lifestyle habits like tobacco smoking. It is necessary to emphasize early education about cancer prevention, especially focused on modification of lifestyle. Adolescents’ understanding of risk factors could help reduce future cancer rates.

## Figures and Tables

**Table 1:** Student’s characteristic.

Characteristic		*n*	%
All students		227	100.0
Age (years)		range: 17–18; median 17.5	
Sex			
	Females	125	55.1
	Males	101	44.5
	no data	1	0.4
Place of residence			
	City	183	80.6
	Village	44	19.4

**Table 2:** The differences in student’s knowledge about cancer due to demographic characteristics.

Examined Factors	Subgroups of Responses	All Students		Sex					Place of Residence				
				Women		Men			City		Village		
		*n*	%	*n*	%	*n*	%	*p* *	*n*	%	*n*	%	*p* *
		227	100	125	55.3	101	44.7		183	80.6	44	19.4	
Correct definition of cancer													
	Yes	179	79	102	81.6	76	75.2	0.37	149	81.5	30	68.2	0.08
	No	46	20	23	18.4	23	22.8		33	18.0	13	29.5	
	no data	2	1			2	2.0		1	0.5	1	2.3	
The most important cancer risk factor													
	Genetic predisposition	153	67.5	94	75.2	59	58.4	0.02	128	70.0	25	56.8	0.10
	Lifestyle **	50	22.0	21	16.8	29	28.7		39	21.3	11	25.0	
	Radiation	23	10.1	9	7.2	13	12.9		15	8.2	8	18.2	
	no data	1	0.4	1	0.8				1	0.5			
People can modify their own cancer risk													
	Yes	183	80.6	101	80.8	81	80.2	0.71	147	81.2	36	81.8	0.93
	No	42	18.5	22	17.6	20	19.8		34	18.8	8	18.2	
	no data	2	0.9	2	1.6				2				
Leading healthy lifestyle													
	Yes	132	58	71	56.8	60	59.4	0.69	110	60.1	22	50.0	0.22
	No	95	42	54	43.2	41	40.6		73	39.9	22	50.0	
Smoking cigarettes													
	Yes, occasionally	45	20	32	25.6	13	12.9	0.13	38	20.8	7	15.9	0.72
	Yes, regularly, a few cigarettes per day	11	5	7	5.6	4	3.9		9	4.9	2	4.5	
	Yes, regularly, >10 cigarettes per day	9	4	4	3.2	5	4.9		8	4.4	1	2.3	
	Yes, electronic	28	12	12	9.6	15	14.9		24	13.1	4	9.1	
	No	134	59	70	56.0	64	63.4		104	56.8	30	68.2	
Smoking status of parents													
	Yes, mother	31	13.6	18	14.4	13	12.9	0.91	26	14.2	5	11.4	0.12
	Yes, father	36	15.9	18	14.4	18	17.8		33	18.0	3	6.8	
	Yes, both parents	24	10.6	13	10.4	10	9.9		21	11.5	3	6.8	
	No	136	59.9	76	60.8	60	59.4		103	56.3	33	75.0	
Is it true that early detected cancer is curable													
	Yes	219	96.5	121	96.8	97	96.0	0.93	178	97.3	41	93.2	0.11
	No	7	3.1	4	3.2	3	3.0		4	2.2	3	6.8	
	no data	1	0.4			1	1.0		1	0.5			

* *p*-value was calculated using chi-square test. ** Lifestyle subgroup included: smoking cigarettes (18.5%), diet (2.2%), low physical activity (0.9%), low socioeconomic status (0.4%), drinking alcohol (0%).

## Data Availability

All data is available at the Department of Oncology at University of Warmia and Mazury in Olsztyn, Poland.

## Supplementary Materials

The following are available online at https://www.mdpi.com/article/10.3390/ijerph18094765/s1, The questionnaire.
