# Supplementary material for: Polish High School Students’ Knowledge about Cancer
Source: Int J Environ Res Public Health. 2021 Apr 29;18(9):4765. doi: 10.3390/ijerph18094765 (PMC8124587; doi:10.3390/ijerph18094765)
Supplement: Supplementary file 1 [file ijerph-18-04765-s001.zip › ijerph-1187329-supplementary.pdf]

1. Wiek: …… lat
2. Płeć
    a. Kobieta
    b. Mężczyzna
3. Miejsce zamieszkania
    a. Miasto
    b. Wieś
4. Co to jest nowotwór?
    a. Choroba, która zawsze prowadzi do śmierci
    b. Oznacza to samo co „rak”
    c. Może być guzem łagodnym lub złośliwym
5. Co według Ciebie w największym stopniu decyduje o zachorowaniu na nowotwory?
    a. Uwarunkowania genetyczne
    b. Niski status społeczny
    c. Palenie papierosów
    d. Picie alkoholu
    e. Dieta
    f. Niska aktywność fizyczna
    g. Promieniowanie
6. Czy według Ciebie mamy wpływ na ryzyko zachorowania na nowotwór?
    a. Tak
    b. Nie
7. Czy uważasz, że prowadzisz zdrowy styl życia?
    a. Tak
    b. Nie
8. Czy palisz papierosy?
    a. Tak, ale sporadycznie
    b. Tak, regularnie, kilka papierosów na dzień
    c. Tak, regularnie, >10papierosów na dzień
    d. Tak, elektroniczne
    e. Nie
9. Czy Twoi rodzice palą?
    a. Tak, mama
    b. Tak, tata
    c. Tak, oboje
    d. Nie
10. Czy uważasz, że wcześnie wykryty nowotwór może być wyleczalny?
    a. Tak
    b. Nie
11. Jak oceniasz swój stan wiedzy na temat chorób onkologicznych?
    a. Bardzo dobry
    b. Dobry
    c. Średni
    d. Słaby
